# An Optical Crack Growth Sensor Using the Digital Sampling Moiré Method

**DOI:** 10.3390/s18103466

**Published:** 2018-10-15

**Authors:** Xinxing Chen, Chih-chen Chang, Jiannan Xiang, Chaobo Zhang, Ming Liu

**Affiliations:** 1 Department of Civil and Environmental Engineering, Hong Kong University of Science and Technology, Hong Kong, China; cechang@ust.hk (C-c.C.); czhangbd@connect.ust.hk (C.Z.); 2 Department of Electronic and Computer Engineering, Hong Kong University of Science and Technology, Hong Kong, China; jxiangad@connect.ust.hk (J.X.); eelium@ust.hk (M.L.)

**Keywords:** optical crack growth sensor, digital sampling moiré, 2D crack growth, calibration, concrete crack

## Abstract

High-accuracy crack growth measurement is crucial for the health assessment of concrete structures. In this work, an optical crack growth sensor using the digital sampling moiré (DSM) method is developed for two-dimensional (2D) crack growth monitoring. The DSM method generates moiré fringes from a single image through digital image processing, and it measures 2D displacements using the phase difference of moiré fringes between motion. Compared with the previous sensors using traditional photogrammetric algorithms such as the normalized cross-correlation (NCC) method, this new DSM-based sensor has several advantages: First, it is of a higher sensitivity and lower computational cost; second, it requires no prior calibration to get accurate 2D displacements which can greatly simplify the practical application for multiple crack monitoring. In addition, it is more robust to the change of imaging distance, which is determined by the height difference between two sides of a concrete crack. These advantages break the limitation of the NCC method and broaden the applicability of the crack growth sensor. These advantages have been verified with one numerical simulation and two laboratory tests.

## 1. Introduction

Concrete deterioration is usually initiated with the appearance of surface cracks. Excessive crack propagation may result in possible dysfunction or even failure of concrete structures [1]. Therefore, crack growth monitoring is of great significance in the risk assessment of a variety of constructed facilities [2]. Currently, a wide range of sensors have been developed to quantitatively measure the crack growth for structural health monitoring purposes. For instance, an optical fiber sensor was imbedded into the structural components to detect and track the opening of a crack on a real bridge [3]. Mao et al. proposed a method for corrosion process monitoring by combining the fiber Bragg grating and Brillouin optical time domain analysis [4]. Li et al. integrated the acoustic emission technique and fiber optic sensing for concrete deterioration tracking [5]. The strain gauges and the linear variable differential transformer (LVDT) were applied to measure the change in the crack width of a concrete specimen in the splitting tension test [6]. The eddy current sensor and the string potentiometer were attached on an adobe house to track the crack extension induced by the vibratory compassion excitation and climatological effects at micro-meter level [7]. These sensors are either imbedded into the structures or mounted on the structural surface, and they are cabled sensors that need to be connected with wires for data transmission. However, cable installation and maintenance are expensive and time-consuming, and the installation of cabled sensors would influence the normal operations of the facilities [8,9].

In contrast, the wireless crack growth sensors transmit data without cables and they have been applied for crack width monitoring on various civil structures: Bennett et al. deployed the wireless sensors with 12 μm resolution for long-term crack monitoring in the Prague Metro and the London Underground [10]; Hoult et al. applied the wireless sensors with a resolution of 10 μm for long-term crack monitoring on bridges [8]; Zhou et al. used a high-accuracy wireless sensors with 1 μm resolution to monitor the dynamic crack width on an in-service reactor containment building during an every-ten years pressure test [9]; Hughi and Marzouk investigated the performance of low-cost piezo-ceramic sensors for crack monitoring [11]; Caizzone and DiGiampaolo developed a passive radio frequency identification (RFID) crack width sensor with submillimeter resolution based on electromagnetic coupling [12].

One problem of these sensors is that they can only measure displacements along the alignment direction of the sensors, while cracks may propagate in multiple directions. Therefore, it is necessary to develop a wireless sensor that can measure two-dimensional (2D) crack growth. Zhang et al. proposed a smart film crack sensor for monitoring the cracks’ location, shape, and propagation [13]. The smart film was composed of enameled wires, and the crack monitoring was based on the proportional relationship between the crack width and ultimate strain of the broken wire. The image processing technique is another full-field method that can track multi-directional crack propagation. Shan et al. presented a stereovision-based method for crack width measurement [14]. Two cameras were used to track the 3D coordinates of two crack edges, and the minimum distance between two edges is regarded as the crack width. Another image processing method, the digital image correlation method, has been applied to assess cracks on fiber concrete beams [15], where a single camera was required that must be aligned well to ensure that the image plane is parallel with the interested region. A “stick and detect” crack growth sensor using the normalized cross-correlation (NCC) method as the image processing algorithm was developed for 2D crack monitoring [16]. This crack growth sensor was glued on one side of the crack and took images of the pattern attached on the other side of the crack. The height difference between the two sides of the crack is the main factor that influences the imaging distance, which is the distance between the sensor and the target. The NCC algorithm tracks the displacement of multiple points in a series of images according to their light intensity distribution [17]. Since the obtained displacement is in the image domain with the unit of pixels, a scale factor (pixel/mm) relating the physical domain and the image domain should be calibrated to calculate the displacement in the physical domain. One challenge is that the scale factor is sensitive to the imaging distance, which is the distance between the sensor and the target [18]. The imaging distances are not constant for all cracks due to their variant height differences between the two sides. Therefore, the scale factor obtained through the calibration process with a fixed imaging distance cannot be ubiquitously applied on uneven cracks with different imaging distances and heights. The mismatch of the scale factor between calibration and application may lead to measurement errors and affect the accuracy of the NCC method [18].

The moiré technique has been adopted for displacement measurement for a long time [19]. Traditionally, the geometric moiré method generates moiré fringes through the overlapping of two regular gratings [20]. The generated moiré fringes can amplify the grating’s motion, so a displacement measurement with a high accuracy can be obtained [19]. A scanning moiré method was proposed to generate moiré fringes by sampling a grating pattern using a scanner composed by a set of regularly spaced dots or lines [21]. However, the sensitivity of the geometric and scanning moiré methods are limited as they only use the information of the moiré fringes’ centerlines [22,23]. To achieve higher sensitivity, a temporal phase shifting method which makes use of the whole intensity profiles of the grating was developed [24,25,26]. A series of moiré fringes were produced by moving the grating with a transducer, and then the obtained moiré fringes were used to compute the phase distribution of the grating. At least three moved moiré fringes are required for the phase calculation of one single grating, therefore this method is not suitable for dynamic analysis. Recently, a digital sampling moiré (DSM) method was developed for high-sensitivity and dynamic displacement measurements [22]. In this method, a series of phase-shifted moiré fringes was generated from one image of the captured grating pattern through down-sampling and up-sampling. The discrete Fourier transform was then used to calculate the phase distribution of the recorded grating. In the final step, the phase difference between the two recorded gratings was used to compute the relative displacement. Results showed that the accuracy of the DSM method could approach 3.8 µm in a three-point bending test of a steel beam, which was equivalent to a 0.01 pixel in the image domain [22]. Comparing this with the NCC method adopted in the wireless crack sensor, one advantage of the DSM method is that the obtained results are in the phase domain, and the corresponding physical displacements can be calculated easily using the predefined pitch length. As a result, the moiré technique does not require any prior calibration to compute the scale factor which relates the image domain with the physical domain, and this technique is robust to the imaging distance since the predefined pitch length is not affected by the imaging distance. 

In this study, an optical crack growth monitoring sensor incorporating the DSM algorithm is developed. This sensor is composed of an optical navigation sensor board (ADNS-3080, Avago technologies, San Jose, CA, USA), a processor (Arduino UNO), a wireless platform (XBee), and a battery. The crack displacements are computed based on a series of images taken by the ADNS-3080. The captured images are processed by the Arduino UNO using the DSM method to calculate the 2D translations of cracks. In the following sections, the development of this sensor is presented, and its performance is tested by the numerical simulation and laboratory tests.

## 2. Prototype and Hardware Components

**Figure 1:**
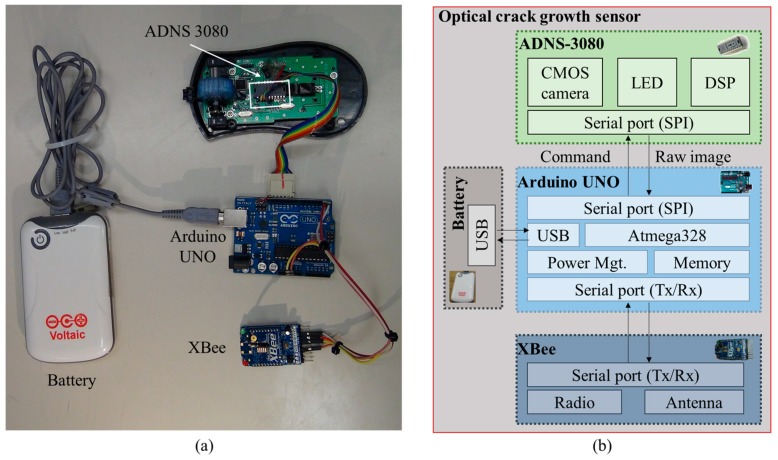
Optical crack growth sensor: (**a**) prototype; (**b**) schematic diagram.

Figure 1a shows a prototype of the optical crack growth sensor, it is composed of four parts: Arduino UNO, ADNS-3080, XBee, and battery. The Arduino UNO is a microprocessor that contains 32 kilobytes (KB) flash memory and 2 KB static random-access memory (SRAM) [27]. The Arduino UNO contains several types of interfaces such as an inter-integrated circuit bus (I2C), a Serial Peripheral Interface Bus (SPI), and a Tx/Rx serial port. The variety of interfaces makes the Arduino UNO adaptable and able to connect with different types of sensors and devices. ADNS-3080 is an optical sensor with a complementary metal-oxide-semiconductor (CMOS) camera which acquires images of the underneath surface illuminated by the embedded light-emitting diode (LED) [28]. It also includes a digital signal processor (DSP) and a serial port. XBee is a wireless communication board with a receiver’s sensitivity as high as −92 dBm [29]. According to the IEEE802.15.4 standard, XBee has a longer communication range than Bluetooth and requires less power [30]. As shown in Figure 1b, the ADNS-3080 and the XBee interface with the Arduino UNO through the Tx/Rx serial port and the SPI port, respectively. The battery can provide power through Universal Serial Bus (USB).

**Figure 2:**
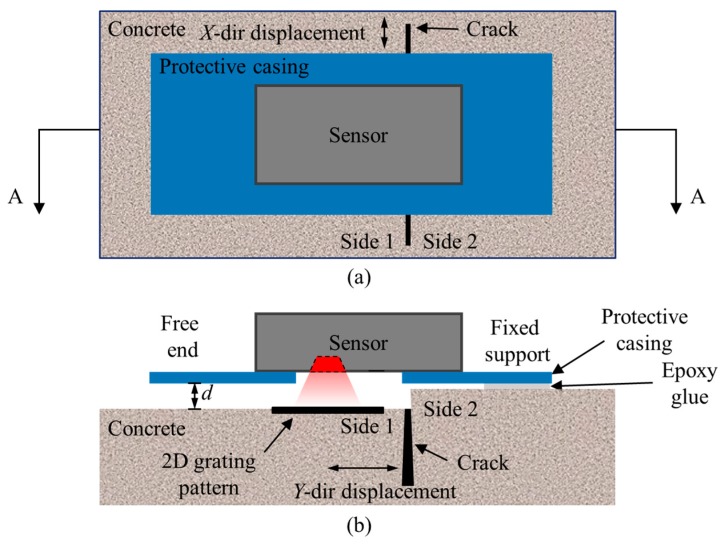
Alignment of the sensor: (**a**) top view; and (**b**) side view of section A-A.

For crack growth monitoring, the ADNS-3080 receives the command from the Arduino UNO to capture images. The captured images are then sent to the Arduino UNO and are processed with the DSM algorithm to calculate 2D displacements. After the calculation, the results are transmitted wirelessly through the XBee to a computer for further analysis. Similar to other crack growth sensors for long-term crack monitoring [8,9,10], this crack growth sensor should be mounted across the crack. As shown in Figure 2, the grating pattern was attached on side 1 of the crack, and it was recorded by the developed sensor fixed on side 2 of the crack. The crack’s growth was measured by detecting the relative motion between the fixed support and the 2D grating pattern. In Figure 2b, *d* is the imaging distance between the sensor and the target pattern. Since the thickness of the epoxy glue used to fix the sensor is ignorable, *d* is mainly determined by the height difference between the two sides of the crack.

## 3. The Digital Sampling Moiré (DSM) Method

**Figure 3:**
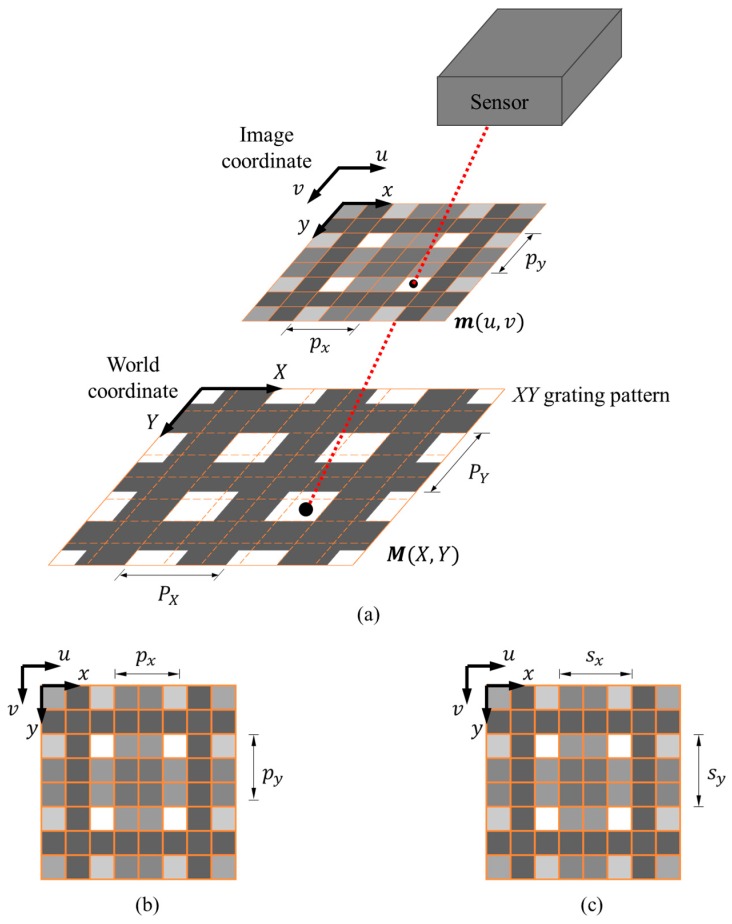
(**a**) The projection of the 2D grating pattern on the image plane; (**b**,**c**) the pitch length ($p_{x}$ and $p_{y}$) and sampling pitch length ($s_{x}$ and $s_{y}$) of the captured 2D grating pattern.

The principle of the DSM method [22] is briefly summarized here. As shown in Figure 3, a 2D grating pattern with *X*- and *Y*-directional gratings is used to generate moiré fringes. The *XY* coordinates are attached on the 2D grating pattern, and their projection on the image plane in the *uv* image coordinates are represented as the *xy* coordinates. The physical lengths of one cycle of the corresponding grating (one white and one black bar) along the *X* and *Y* directions are denoted as $P_{X}$ and $P_{Y}$, respectively, and their corresponding pitch lengths in the image domain are denoted as $p_{x}$ and $p_{y}$, respectively. As shown in Figure 3b,c, the nearest integer values of pitch lengths of *X*- and *Y*-directional gratings in the image plane are $s_{x}$ and $s_{y}$, respectively. To simplify the following derivation, $P_{X}$ and $P_{Y}$ are set to be identical as *P*, and likewise $p_{x}=p_{y}=p$, and $s_{x}=s_{y}$ = s. 

**Figure 4:**
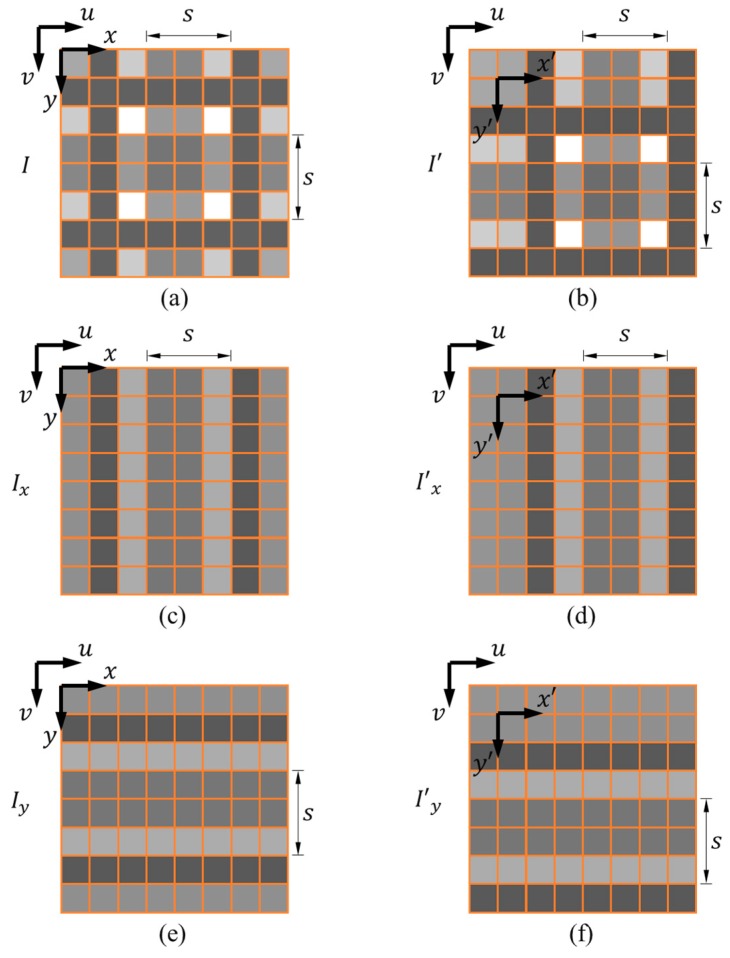
The captured 2D grating pattern in (**a**) the reference image I and (**b**) the subsequent image I’; the extracted (**c**) *x*-directional grating $I_{x}$ and (**d**) x’-directional grating ${I’}_{x}$; the extracted (**e**) *y*-directional grating $I_{y}$ and (**f**) y’-directional grating ${I’}_{y}$.

**Figure 5:**
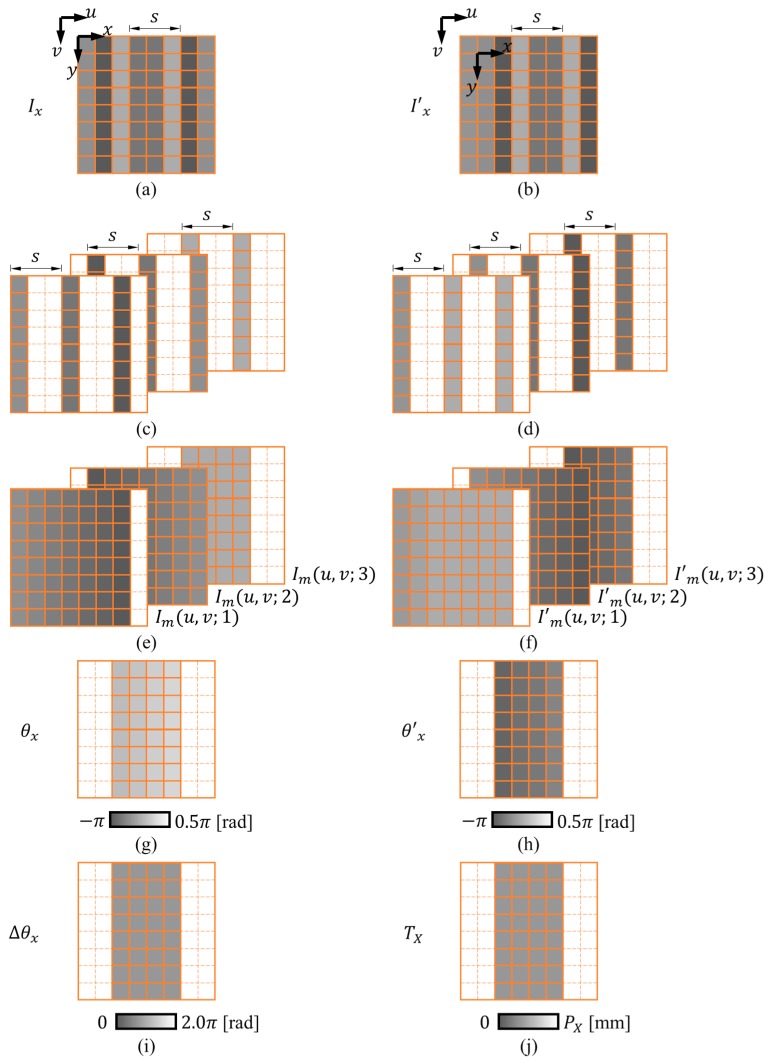
The calculation process of *x*-directional displacement $T_{X}$ using the digital sampling moiré (DSM) method. (**a**,**b**) The filtered *x*-directional grating between motion; (**c**,**d**) corresponding down-sampled gratings; (**e**,**f**) corresponding moiré fringes; (**g**,**h**) phase distribution of corresponding moiré fringes; (**i**) the phase difference between the motion; (**j**) the obtained $T_{X}$.

The DSM method computes the relative 2D displacement using a reference image *I* and a subsequent image I’ acquired between the motion of the grating pattern (see Figure 4a,b). The first step is to extract the *x*- and *y*-directional gratings using s × 1 pixel and 1 × s pixel spatial average filters, respectively. The *x*-directional grating $I_{x}$ and $x^{\prime }$-directional gratings ${I’}_{x}$ filtered from I and I’ are shown in Figure 4c,d, respectively. The *y*-directional grating $I_{y}$ and y’-directional gratings ${I’}_{y}$ filtered from I and I’ are shown in Figure 4e,f, respectively. The calculation process of *X*-directional displacement $T_{X}$ is demonstrated in Figure 5. The extracted *x*-directional grating $I_{x}(u,\;v)$ and ${I’}_{x}(u,\;v)$ are shown in Figure 5a,b, respectively. Express $I_{x}(u,\;v)$ as

(1) $$I_{x}{(u,\;v)\;=\;a(u,\;v)\cos[\varphi }_{x}(u,\;v)]\;+\;b(u,\;v),$$

where a(u, v) is the amplitude of the grating intensity, and b(u, v) represents the background intensity, and $\varphi_{x}(u,\;v)$ is the phase distribution of the *x*-directional grating.

The DSM method generates moiré fringes through down-sampling and interpolation. Figure 5c,d illustrate the down-sampling of the gratings $I_{x}$ and ${I’}_{x}$, in which every s pixels (in this illustration, s = 3 pixels) are chosen starting from the 1st, 2nd, and 3rd pixels to obtain a series of down-sampled gratings, respectively. Next, interpolation is performed to generate the corresponding moiré fringes shown in Figure 5e,f. The following equation presents the *k*-th moiré fringe $I_{m}\;(u,\;v;\;k)$

(2) $$I_{m}{(u,\;v;\;k)\;=\;a\;(u,\;v)\cos[\theta }_{x}(u,\;v)\;+\;\frac{2\pi \;(k-1)}{s}]+\;b\;(u,\;v),\;(k\;=\;1,\;\ldots .\;,\;s)\;$$

where $\theta_{x}(u,\;v)$ is the phase distribution of the first moiré fringe, as shown in Figure 5g. Using the discrete Fourier transform, its value can be calculated as,

(3) $$\theta_{x}(u,\;v)\;=\;-arctan\frac{\sum_{k=1}^{s}I_{m}\;(u,\;v;\;k)\;\times \;\sin(\frac{2\pi k}{s})}{\sum_{k=1}^{s}I_{m}\;(u,\;v;\;k)\;\times \;\cos(\frac{2\pi k}{s})},$$

Following the same procedure, the phase distribution of the moiré fringe after motion ${\theta ’}_{x}(u,\;v)$ can be obtained by utilizing the moiré fringes ${I’}_{m}(u,\;v;\;k)$ (Figure 5h). Next, the phase difference $\Delta \theta_{x}$ (Figure 5i) can be calculated from the following equation:

(4) $$\Delta \theta_{x}{(u,\;v)\;=\;\theta }_{x}{(u,\;v)\;-\;\theta ’}_{x}(u,\;v).$$

Finally, knowing the phase difference, the translation along *X*-direction, $T_{X}\;(u,\;v)$ (Figure 5j), can be calculated as follows

(5) $$T_{X}(u,\;v)\;=\;\Delta \theta_{x}\;(u,\;v)\;\times \;\frac{P}{2\pi },$$

the translation along *Y*-direction $T_{Y}\;(u,\;v)$ can be obtained in the same way.

## 4. Performance Tests of the Optical Crack Growth Sensor

### 4.1. Simulation

**Figure 6:**
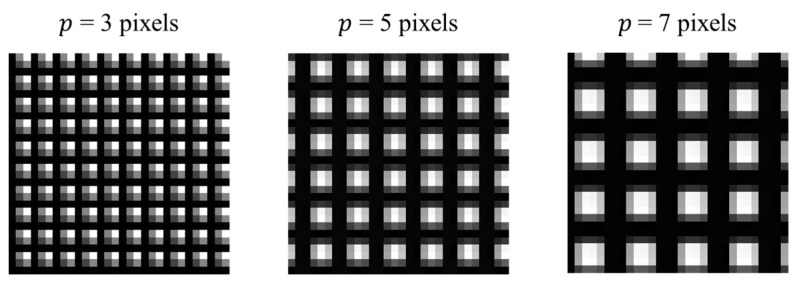
Simulation: the 2D grating patterns with different pitch lengths.

In a previous study, the NCC method was embedded in an optical crack growth sensor to measure 2D crack displacements [16]. In this simulation, the DSM method was compared with the NCC method in terms of efficiency and accuracy. The NCC and DSM methods were coded in C language and uploaded to the Arduino UNO to process the same set of simulated images. The images were generated by MATLAB with a resolution of 24 × 24 pixels, corresponding to an area of 1.4 × 1.4 mm in the physical domain. The images were programmed to move at 10 µm/step for 10 steps. Different sets of simulated images with pitch lengths ranging from 3 to 7 pixels were analyzed in this simulation. Figure 6 shows the three representative 2D grating patterns with pitch lengths equal to 3, 5, and 7 pixels.

The performance of the NCC method is determined by the size of the region of interest (ROI) and the searching step for correlation calculation. A larger ROI produces more stable results and smaller searching step leads to higher sensitivity. On the other hand, the improved accuracy will result in a higher computational cost [16]. Following the previous study, the size of ROI was set to be 6 × 6 pixels and the searching step was equal to 0.2 pixel [16]. The simulated images were processed with the NCC algorithm embedded in the crack growth sensor, and the computational time for one output was 1.61 s. For the DSM method, its computational cost is related to the pitch length (*p*). When *p* ranges from 3 to 7 pixels, the corresponding computational cost ranges from 1.42 to 1.59 s per output, which is smaller than that of the NCC method.

**Figure 7:**
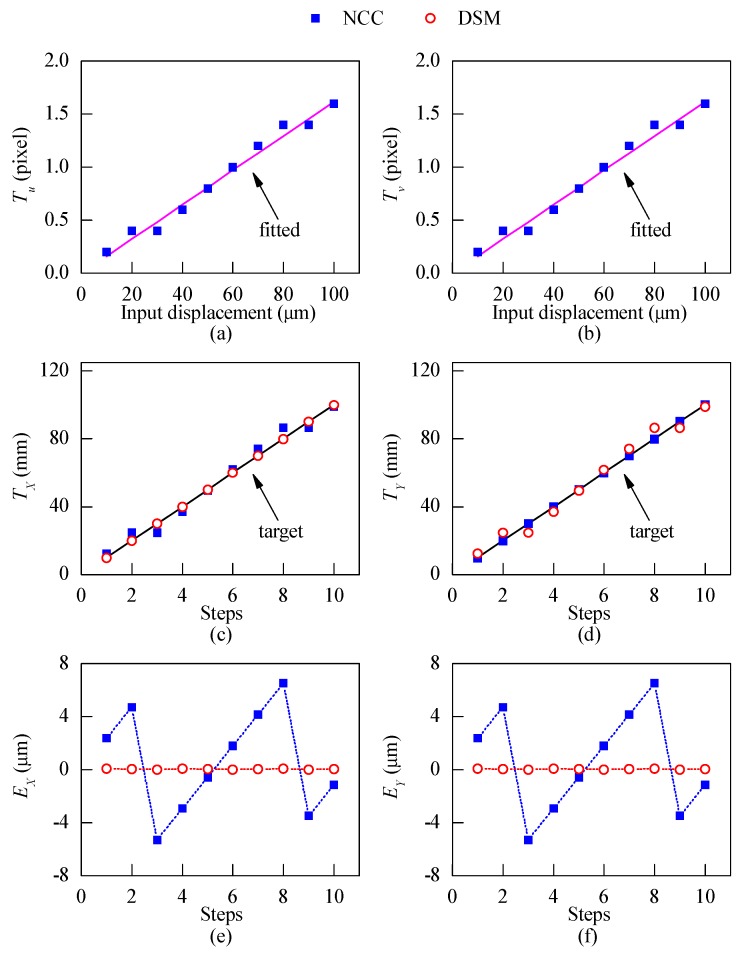
(**a**,**b**) Calibration for the normalized cross-correlation (NCC) method; (**c**,**d**) displacements obtained by the NCC and the DSM method; (**e**,**f**) displacement errors of two methods in the simulation.

Since the displacements obtained by the NCC method are in the image domain with pixel dimensions, a scale factor should be used to convert the displacements to the physical domain. Therefore, prior to the displacement measurement using the NCC method, calibration should be performed to obtain the scale factor [18]. Figure 7a,b show the measured displacements in pixel dimensions along the *u*- and *v*-direction, $T_{u}$ and $T_{v}$ obtained by the NCC method when *p* = 3 pixels, respectively. A line was fitted to the results using the least square method. The slopes of the fitted lines are the scale factors between the image coordinates and the physical coordinates. The average scale factor for two directions was found to be 16.2 pixels/mm. 

In the DSM method, as shown in Equation (5), the phase difference of moiré fringes can be converted to the displacement in the physical domain with a predefined pitch length, hence no calibration is required. Figure 7c,d show the displacements in the physical domain obtained by the NCC method and the DSM method, respectively. It was observed that both methods can track the displacements along two directions. To compare the accuracy of the two methods, their errors were defined as the difference between the calculated and the actual displacements ($T_{\mathrm{calculate}}-T_{\mathrm{true}}$) and plotted in Figure 7e,f. The errors of the DSM method are significantly smaller than those of the NCC method. The zigzag pattern of the error distribution of the NCC method is due to the limitation of its resolution. The resolution of the NCC method is mainly determined by the searching step [16], which was 0.2 pixel in this case. As the calibrated scale factor is 16.2 pixel/mm, the resolution of the NCC method is 12.5 µm. The mean absolute error (MAE) and standard deviation (SD) are used here to quantify the error. For the DSM method, the MAE and SD are 0.04 μm and 0.03 µm, respectively. In comparison, the MAE and SD of the NCC method are 3.41 µm and 3.90 µm, respectively. Hence, for *p* = 3 pixels, the accuracy of the DSM method is about 100 times higher than the NCC method.

**Figure 8:**
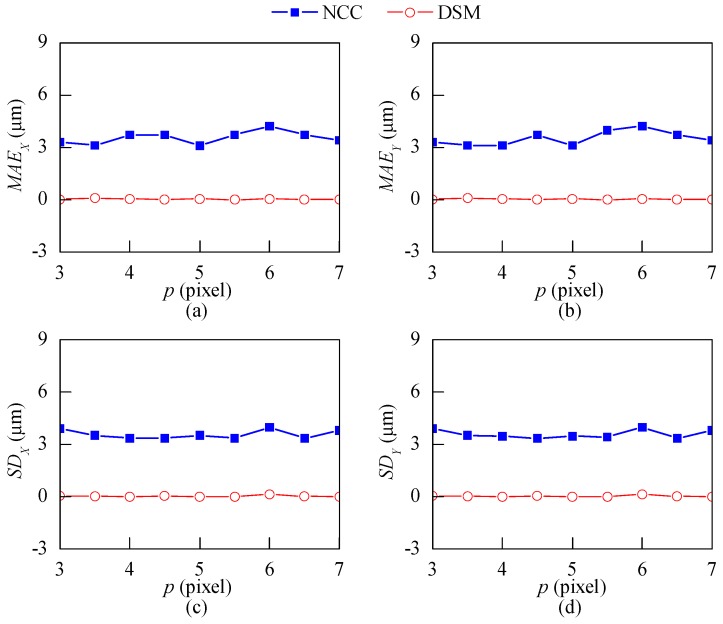
Error analysis for patterns with different pitch lengths. (**a**,**b**) The mean absolute error (MAE); (**c**,**d**) standard deviation (SD).

Figure 8 shows the error analysis of the two methods when *p* changes from 3 to 7 pixels with an interval of 0.5 pixels. The MAE and SD of the NCC method range from 3 µm to 4 µm, while the corresponding values of the DSM method are smaller than 0.12 µm. This numerical simulation illustrates that the NCC method requires calibration to obtain the scale factor, which is not required by the DSM method. Moreover, the DSM method is of higher accuracy than the NCC method. 

### 4.2. The XYZ-Table Test

In this section, to demonstrate the accuracy of the DSM method and its robustness to the change of imaging distance, an *XYZ*-table test was performed.

**Figure 9:**
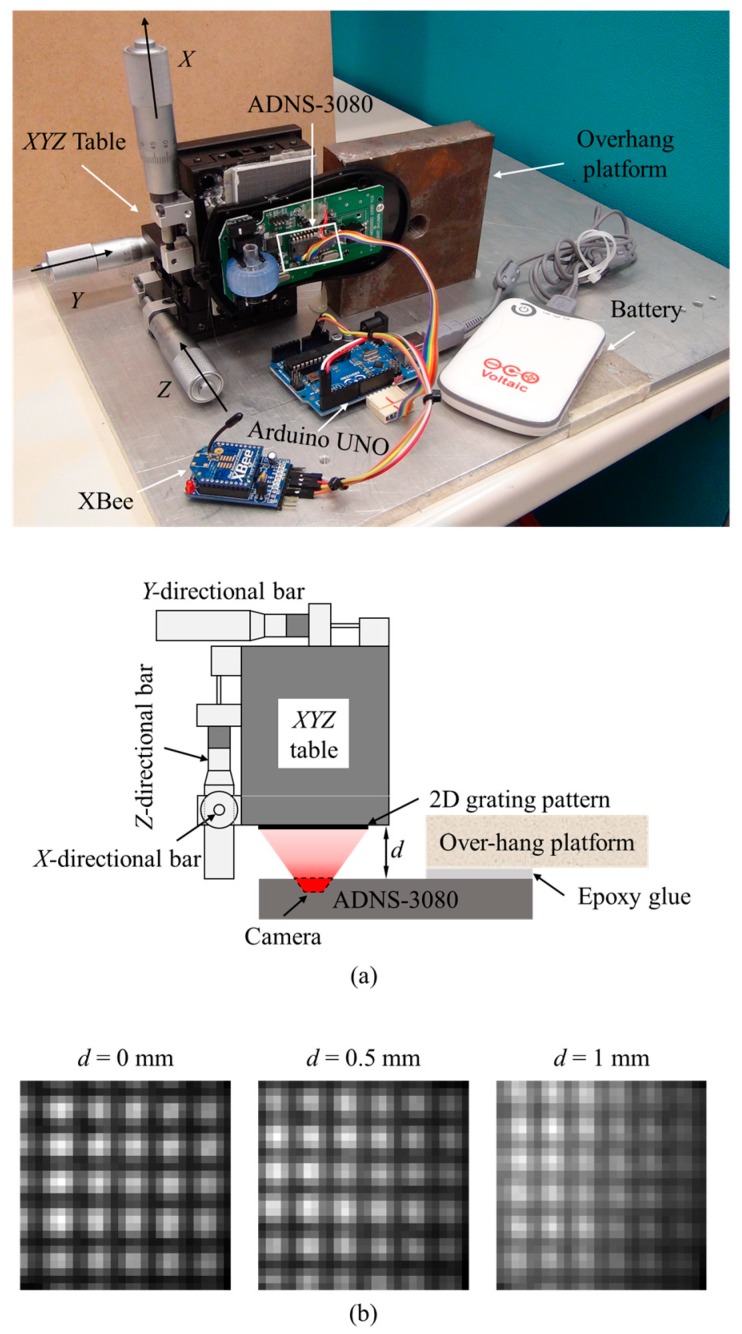
The *XYZ*-table test: (**a**) schematic of the setup; (**b**) the captured 2D grating patterns at different distances (*d*).

The setup of *XYZ*-table test is shown in Figure 9a. In brief, ADNS-3080 was mounted on a fixed over-hang platform as a cantilever support on one side, while the camera of the ADNS-3080 was targeted at the 2D grating pattern attached onto the *XYZ*-table. The images captured by the ADNS-3080 would be sent to Arduino UNO and XBee for analysis and wireless transmission. The *XYZ*-table can be manually controlled to translate along three directions with an accuracy of 1 µm. The relative 2D motion between the fixed over-hang platform and the *XYZ*-table can be adjusted by the *X*- and *Y*-directional bars to simulate 2D crack growth. The *Z*-directional bar controls the distance (*d*) from the optical crack growth sensor and the target, which was used to simulate the variation of *d* for monitoring cracks with different relative heights between two surfaces (Figure 2b).

In this test, similar to the previous part, the NCC method and the DSM method were used to measure the displacement of the grating pattern attached to the *XYZ*-table. As mentioned before, the NCC method requires calibration to get the scale factor relating the physical domain and the image domain. The calibration process for the NCC method was performed in a similar way as described in the previous study [16]. During the calibration, the sensor is usually kept very close to the target, while no friction was allowed between them (*d* = 0 mm). After the calibration, the crack growth sensor is installed on a real crack to measure crack displacements with the scale factor obtained in the calibration. As mentioned earlier, the scale factor is a function of the distance *d*. For a real case with a different height of the concrete surfaces on the two sides of an uneven crack (Figure 2b), it is likely that there would be a discrepancy between the calibration and the actual condition. Therefore, the inconsistency of the scale factor between the calibration and the real application could be a source of measurement errors for the NCC method. On the other hand, as shown in Equation (5), the DSM method obtains the displacement in the physical domain using the predefined value P that is independent of the distance *d*. Therefore, the accuracy of the DSM method is less sensitive to the distance *d*.

In this *XYZ*-table test, the robustness of the DSM and the NCC methods to the *d* were studied. As shown in Figure 9a, *d* can be controlled by rotating the *Z*-directional bar of the *XYZ*-table. In the test, *d* values were controlled to be 0 mm, 0.5 mm, and 1 mm, and the captured images of the 2D grating patterns for different *d* values are shown in Figure 9b. It is found that the captured 2D grating pattern contains more gratings with the increase of *d*. The enlarged field of view is due to the larger scale factor (mm/pixel) with the increase of *d*.

Calibration was performed for *d* = 0 mm by moving the 2D grating pattern along *X*- and *Y*-directions simultaneously with 10 µm per step for 10 steps in total. Linear fitting was used to get the scale factor based on the input displacements and the output $T_{u}$ and $T_{v}$. The scale factor was calculated to be 16.7 pixel/mm. After calibration, for each *d* value (*d* = 0 mm, 0.5 mm, or 1.0 mm), the target pattern was controlled to translate in 2D with 10 µm per step along *X*- and *Y*-directions.

**Figure 10:**
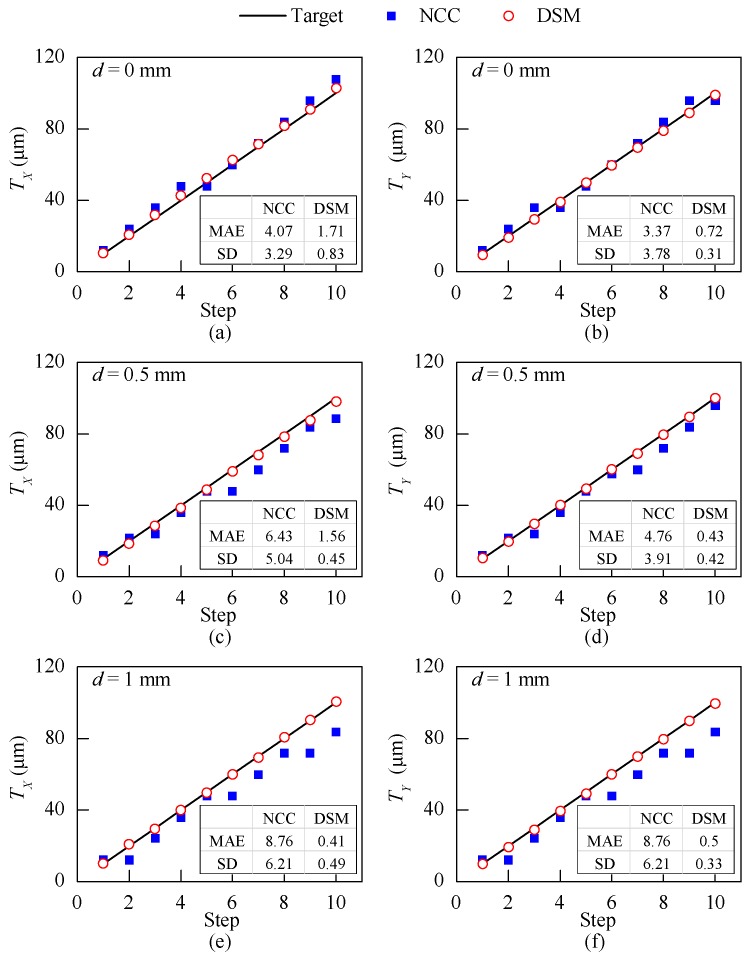
The *XYZ*-table test: the *X*- and *Y*-direction displacement measurement results when (**a**,**b**) *d* = 0 mm*,* (**c**,**d**) *d* = 0.5 mm and (**e**,**f**) *d* = 1.0 mm.

The captured grating images with the different *d* values were analyzed by both the DSM and the NCC methods, and the results are plotted in Figure 10. It shows that the DSM method tracks the displacements precisely for the three values of *d*. On the contrary, the errors of the NCC method increased as *d* changes from 0 mm to 1 mm. The increased errors mainly result from the change of scale factor when *d* alters. The MAE and SD of the measurement results with the two methods for each value of *d* are also shown in Figure 10. The error of the DSM method is not affected by *d* and its MAE and SD remain smaller than 2 µm and 1 µm, respectively. In contrast, for the NCC method, the MAE and SD of measurements increase from 3.37 µm to 8.76 µm and from 3.29 µm to 6.21 µm, respectively, as *d* changes from 0 mm to 1 mm.

This laboratory *XYZ*-table test shows that the DSM method can achieve higher accuracy than the NCC method, while needs no prior calibration. Moreover, it is verified that the DSM method is more robust to the change of *d* (see Figure 2). The NCC method requires a calibrated scale factor (mm/pixel) to convert the 2D displacement from pixel domain ($T_{u}$ and $T_{v}$) to the physical domain ($T_{X}$ and $T_{Y}$). The results presented in Figure 10 show that the DSM method is more robust to the change of *d* than the NCC method. This is because the NCC requires a calibrated scale factor (mm/pixel) to convert the 2D displacement from pixel domain ($T_{u}$ and $T_{v}$) to the physical domain ($T_{X}$ and $T_{Y}$). This scale factor is affected by *d*, hence different *d* values would lead to different measurement results. On the other hand, the DSM method measures the physical displacement using a predefined pitch length *P* which is input to the algorithm as a constant. The robustness to imaging distance makes the DSM method especially suitable to measure the propagation of uneven cracks in which the distance between sensor and crack surface is difficult to control.

### 4.3. Concrete Crack Test

**Figure 11:**
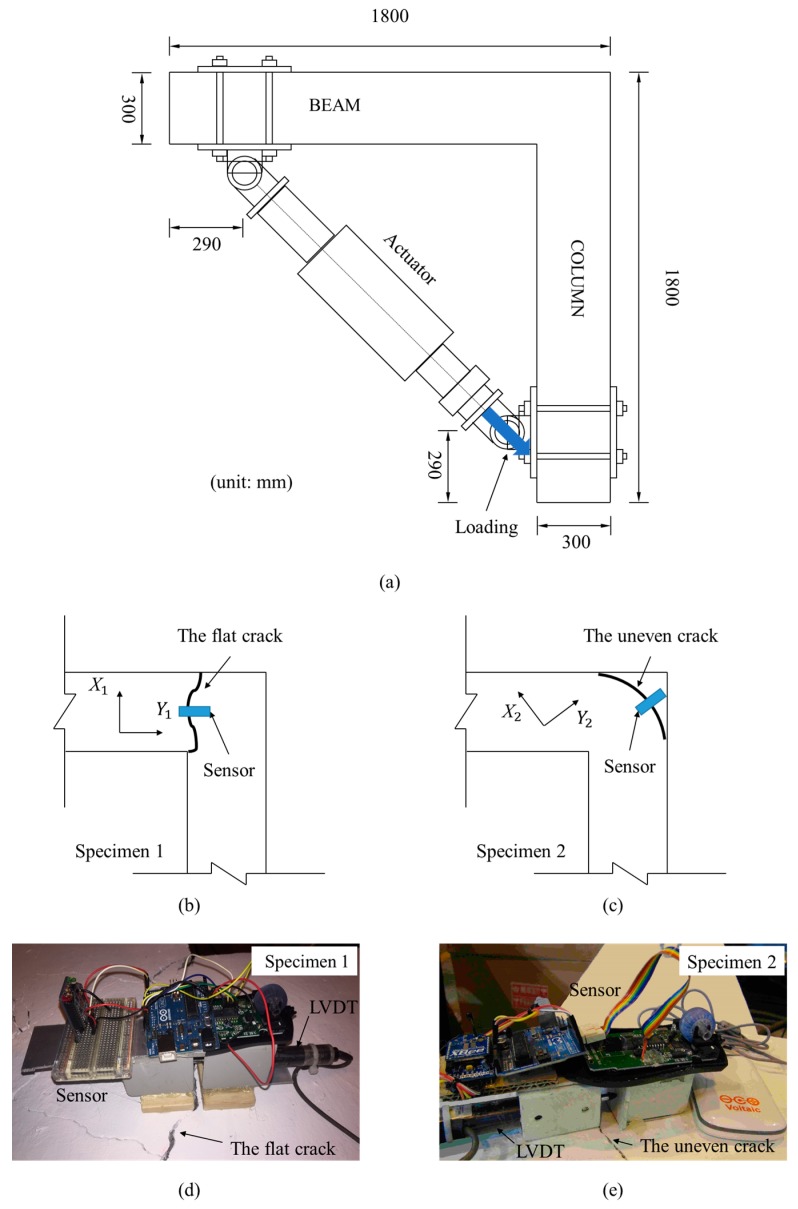
The beam-column knee joint test: (**a**) the vertical view of the RC specimens; schematics of (**b**) the 1st crack and (**c**) the 2nd crack; the alignment of the sensors on (**d**) the 1st crack and (**e**) the 2nd crack.

To evaluate the performance of the developed optical crack growth sensor for real crack monitoring, a reinforcement concrete (RC) structure test was conducted to measure the motions of two cracks with different *d* values: a flat crack (*d* = 0 mm) and an uneven crack (*d* > 0 mm). The cracks were located on two RC beam-column knee joints. Figure 11a shows the top view of the specimens. The lengths of the beams and the columns for both specimens were 1800 mm. For Specimen 1, the beam and the column had the same square cross-section of 300 mm × 300 mm, while the corresponding cross-sections of Specimen 2 were 300 mm × 300 mm and 300 mm × 400 mm, respectively. A diagonally placed hydraulic actuator was connected to the beam and column tips of the specimens to produce harmonic motion. Specimen 1 and Specimen 2 were forced by the actuator to move with 3 mm and 30 mm amplitude, respectively, under the frequency of 0.01 Hz. Figure 11b,c show the location of the considered cracks on Specimen 1 and Specimen 2, respectively. The crack on Specimen 1 was flat, while the crack on Specimen 2 was uneven and its *d* value approached 1.5 mm. The optical crack growth sensor was attached across the cracks on two specimens to monitor their harmonic motions, as shown in Figure 11d,e. Apart from the optical sensor, an LVDT with 5 µm resolution was installed beneath the sensor to measure the one-dimensional displacement of the cracks, as shown in Figure 11d,e. The LVDT measured $Y_{1}$- and $Y_{2}$-directional motion for the 1st and the 2nd crack, respectively.

**Figure 12:**
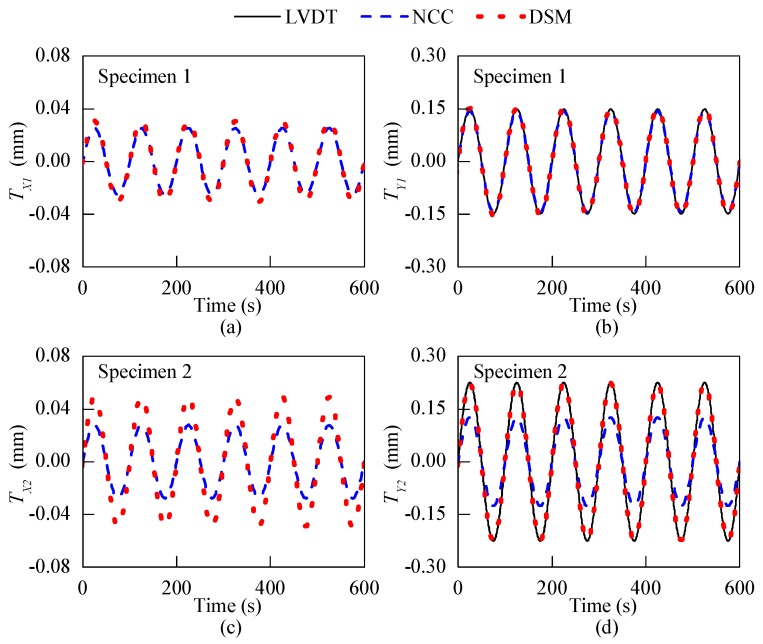
The beam-column knee joint test: displacement responses of cracks on two specimens along *X*- and *Y*-direction.

The results obtained by the LVDT and the optical sensor on two cracks are plotted in Figure 12. The NCC and DSM algorithms were integrated into the optical crack growth sensor, respectively, to compare with the LVDT. Figure 12a,b show the $X_{1}$- and $Y_{1}$-directional displacements of the 1st crack, in which the displacements obtained by the DSM and the NCC methods match well with each other. In Figure 12b, the $Y_{1}$-directional displacement measured by the LVDT shows a good agreement with the other two methods. However, Figure 12c,d show that for the second specimen the displacements obtained by the NCC method are significantly smaller than those measured by the DSM method. Besides, as shown in Figure 12d, the displacements measured by the LVDT agree well with those of the DSM method, indicating that only the DSM method can accurately track the propagation of cracks with uneven surfaces. The lower accuracy of the NCC method is due to the mismatch of the scale factor between the calibration and real application. As the calibration was performed for *d* = 0 mm, the uneven surfaces of the crack increase the *d* value and make the calibrated scale factor inaccurate for real application.

This laboratory test on real cracks demonstrates the advantages of the DSM method to measure the displacements of uneven cracks with high accuracy. This calibration-free and robust DSM-based crack growth sensor can play an important role in the crack growth monitoring on a variety of civil structures.

## 5. Discussion

In this work, a DSM-based optical crack growth sensor for the health assessment of concrete structures was developed by integrating Arduino UNO, XBee, and an optical navigation sensor board (ANDS-3080). Compared to the previous sensor which uses the NCC method as displacement measurement algorithm, this newly developed sensor adopts the DSM method and exhibits a few improvements. First, a calibration process is not required to get the accurate scale factor between the image domain and the physical domain; second, it can be applied on cracks with different *d* (the imaging distance between the target pattern and the sensor); third, a higher accuracy can be achieved with a lower computational cost.

Simulations and laboratory tests were performed to compare the NCC and DSM methods in multiple aspects. The simulation shows that the DSM method does not need calibration to obtain the scale factor of the imaging system, and it can achieve a higher sensitivity at a lower computational cost than the NCC method. The *XYZ*-table test demonstrates that the DSM-based sensor can achieve high-accuracy displacement measurements with MAE and SD smaller than 2 μm and 1 μm, respectively. Meanwhile, it also shows the robustness of the DSM method to the imaging distance *d*. The optical sensor was then used to track the 2D harmonic motion of two real cracks. The results show that the NCC method can measure accurate displacements only for the crack with a flat surface, of which the scale factor is consistent between calibration and the real test. On the other hand, the DSM-based sensor can detect precise crack motions for both flat and uneven cracks with different *d* values. The results exhibit the robustness of the proposed optical sensor to uneven cracks on real structures. These advantages make the DSM-based optical crack growth sensor a powerful tool for high-accuracy monitoring of 2D concrete crack growth.

While the experimental tests in this proof-of-concept study were performed in a laboratory under relatively stable temperature and humidity conditions, the proposed sensor exhibits some characteristics which might give it some edges for actual implementation. The operating temperature of the sensor’s main components (ADNS-3080, Arduino UNO, XBee, and battery) is in the range of 0–40 °C, which seems to meet the demand of most structures under normal environmental conditions. In addition, this sensor has an embedded camera and a built-in LED as light source. In application, the sensor is attached closely to the concrete surface and the lighting for image acquisition is solely supplied by the built-in LED. Its fixed mechanical structure creates a stable illumination and image acquisition condition that minimizes the influence of ambient light. However, the sensor’s components should be condensed, integrated, and weather-proofed before the sensor can be used for long-term monitoring under ambient conditions. Moreover, the temperature change would induce the deformation of the concrete and the grating pattern. To reduce the influence of this, the concrete deformation caused by thermal effects should be compensated for, and the grating pattern could be printed on a solid material such as a high-resistance magnet [30].

## References

[1] Mohammad I., Huang H. (2010). Monitoring fatigue crack growth and opening using antenna sensors. Smart Mater. Struct..

[2] Hoult N.A., Dutton M., Hoag A., Take W.A. (2016). Measuring crack movement in reinforced concrete using digital image correlation: Overview and application to shear slip measurements. Proc. IEEE.

[3] Enckell M., Glisic B., Myrvoll F., Bergstrand B. (2011). Evaluation of a large-scale bridge strain, temperature and crack monitoring with distributed fibre optic sensors. J. Civ. Struct. Health Monit..

[4] Mao J., Chen J., Cui L., Jin W., Xu C., He Y. (2015). Monitoring the corrosion process of reinforced concrete using BOTDA and FBG sensors. Sensors.

[5] Li W., Xu C., Ho S.C.M., Wang B., Song G. (2017). Monitoring concrete deterioration due to reinforcement corrosion by integrating acoustic emission and FBG strain measurements. Sensors.

[6] Rapoport J., Aldea C., Shah S.P., Ankenman B., Karr A. (2002). Permeability of cracked steel fiber-reinforced concrete. J. Mater. Civ. Eng..

[7] Abeel P., Aimone-Martin C., Dowding C. Autonomous crack measurement for comparison of vibratory compaction excitation and climatological effects. Proceedings of the 7th European Workshop on Structural Health Monitoring (EWSHM).

[8] Hoult N.A., Fidler P.R., Hill P.G., Middleton C.R. (2010). Long-term wireless structural health monitoring of the Ferriby Road Bridge. J. Bridg. Eng..

[9] Zhou J., Xu Y., Zhang T. (2016). A wireless monitoring system for cracks on the surface of reactor containment buildings. Sensors.

[10] Bennett P.J., Soga K., Wassell I., Fidler P., Abe K., Kobayashi Y., Vanicek M. (2010). Wireless sensor networks for underground railway applications: Case studies in Prague and London. Smart Struct. Syst..

[11] Hughi D., Marzouk H. (2015). Crack width monitoring system for reinforced concrete beams using piezo-ceramic sensors. J. Civ. Struct. Health Monit..

[12] Caizzone S., DiGiampaolo E. (2015). Wireless passive RFID crack width sensor for structural health monitoring. IEEE Sens. J..

[13] Zhang B., Wang S., Li X., Zhang X., Yang G., Qiu M. (2014). Crack width monitoring of concrete structures based on smart film. Smart Mater. Struct..

[14] Shan B., Zheng S., Ou J. (2016). A stereovision-based crack width detection approach for concrete surface assessment. KSCE J. Civ. Eng..

[15] Hamrat M., Boulekbache B., Chemrouk M., Amziane S. (2016). Flexural cracking behavior of normal strength, high strength and high strength fiber concrete beams, using digital image correlation technique. Constr. Build. Mater..

[16] Man S.H., Chang C.C. (2016). Design and performance tests of a LED-based two-dimensional wireless crack propagation sensor. Struct. Control Health Monit..

[17] Yoo J.C., Han T.H. (2009). Fast normalized cross-correlation. Circuits Syst. Signal Process.

[18] Palacin J., Valganon I., Pernia R. (2006). The optical mouse for indoor mobile robot odometry measurement. Sens. Actuators A Phys..

[19] Walker C.A. (2003). Handbook of Moiré Measurement.

[20] Yokozeki S., Kusaka Y., Patorski K. (1976). Geometric parameters of moiré fringes. Appl. Opt..

[21] Morimoto Y., Hayashi T. (1984). Deformation Measurement during powder compaction by a scanning-moire Method. Exp. Mech..

[22] Ri S., Fujigaki M., Morimoto Y. (2010). Sampling moiré method for accurate small deformation distribution measurement. Exp. Mech..

[23] Wang Q.H., Ri S., Tsuda H., Koyama M., Tsuzaki K. (2017). Two-dimensional moiré phase analysis for accurate strain distribution measurement and application in crack prediction. Opt. Express.

[24] Choi Y.B., Kim S.W. (1998). Phase-shifting grating projection moiré topography. Opt. Eng..

[25] Kujawinska M. (1987). Use of phase-stepping automatic fringe analysis in moiré interferometry. Appl. Opt..

[26] Ai C., Wyant J.C. (1987). Effect of piezoelectric transducer nonlinearity on phase shift interferometry. Appl. Opt..

[27] Arduino UNO REV3. https://store.arduino.cc/usa/arduino-uno-rev3.

[28] ADNS 3080. https://people.ece.cornell.edu/land/courses/ece4760/FinalProjects/s2009/ncr6_wjw27/ncr6_wjw27/docs/adns_3080.pdf.

[29] XBee^®^/XBee-PRO^®^ RF Modules. https://www.sparkfun.com/datasheets/Wireless/Zigbee/XBee-Datasheet.pdf.

[30] Lee J.S., Su Y.W., Shen C.C. A comparative study of wireless protocols: Bluetooth, UWB, ZigBee, and Wi-Fi. Proceedings of the 33rd Annual Conference of IEEE Industrial Electronics Society (IECON).

